# Happiness and depression in psoriasis: a cross-sectional study in Germany

**DOI:** 10.1007/s11136-021-02991-2

**Published:** 2021-09-16

**Authors:** Barbara Schuster, Corinna Peifer, Stefanie Ziehfreund, Linda Tizek, Tilo Biedermann, Alexander Zink, Maximilian C. Schielein

**Affiliations:** 1 Department of Dermatology and Allergy, School of Medicine, Technical University of Munich, Munich, Germany; 2 Institute for Medical Information Processing, Biometry, and Epidemiology (IBE), Pettenkofer School of Public Health, LMU Munich, Munich, Germany; 3 Department of Psychology, University of Lübeck, Lübeck, Germany; 4 Division of Dermatology and Venereology, Department of Medicine, Karolinska Institutet, Stockholm, Sweden

**Keywords:** Psoriasis, Happiness, Subjective well-being, Depression, Mental health, Dermatology

## Abstract

**Purpose:**

Prior research on the psychological consequences of skin diseases has focused on assessing mental comorbidities. The aim of this study was to investigate subjective well-being in a large sample of individuals affected by psoriasis, a chronic inflammatory skin disease, and to explore the associations with depression and disease-related parameters such as disease severity.

**Methods:**

A cross-sectional online survey was conducted from March to June 2019. The link to the questionnaire was shared on websites and Facebook pages of psoriasis patient organizations and campaigns. Participants filled in validated scales measuring subjective well-being—operationalized as positive affect (PA), negative affect (NA) and satisfaction with life (SWL); and depression.

**Results:**

The data of 722 participants were analyzed. Exploratory factor analysis supported the differentiation of PA, NA, SWL, and depression as four different constructs. The respondents reported lower levels of PA than healthy individuals and judged themselves to be less happy and were less satisfied with their lives than the general population (except age group 65 + years). 40.3% of respondents were screened positive for depression. More severe psoriasis was associated with lower affective well-being and a higher risk for depression.

**Conclusion:**

The results of this study empirically supported the differentiation of subjective well-being and depression as different constructs in individuals with psoriasis, and underline the large mental burden of the disease which goes beyond a higher risk for depression. Measures of well-being should thus be incorporated in both research and clinical practice in patients with psoriasis in order to achieve a more comprehensive picture of the mental burden of this disease.

## Plain English summary

Chronic skin diseases such as psoriasis can be a large mental burden for the affected individuals. Previous research has focused on assessing mental comorbidities such as depression in order to determine mental health in patients with skin diseases. However, according to the World Health Organization, there is more to being well than just not being ill. Thus, in order to fully capture the mental burden of skin diseases, both mental comorbidities and well-being need to be considered. Therefore, the aim of this study was to assess the mental burden of patients with psoriasis by not only measuring depression, but also happiness. We found that patients with psoriasis are less happy and have a higher risk for depression than healthy individuals. Treating skin symptoms can help the patients to be happier and less depressed. However, additional psychological care should be provided to patients with severe psoriasis in order to improve the patients’ well-being.

## Introduction

Psoriasis is a common chronic inflammatory skin disease with an estimated prevalence of 1.2–3% in Germany [1, 2]. It is characterized by sharply demarcated red patches covered in typical whitish-silver scales, typically, but not only, located on knees and elbows as well as the scalp [3]. A large number of patients suffer from severe itch, and some of them, even pain [4, 5]. In addition, psoriasis is associated with a large number of comorbidities, ranging from arthritis and metabolic syndrome to depression [3, 6, 7]. As psoriatic lesions are often considered disfiguring by their social environments, many affected patients experience body image issues [8] and suffer from stigmatization and social exclusion [9, 10], resulting in a large psychological disease burden [7, 11, 12]. As a consequence, psoriasis has been recognized as a serious noncommunicable disease by the World Health Assembly in 2014 [13].

### Assessing the mental burden of psoriasis

Prior research on the psychological consequences of skin diseases has predominantly taken a pathogenic perspective, with most studies exploring mental comorbidities as markers of mental health. The results in general indicated a higher risk of depression, anxiety, and addiction for patients with psoriasis [6, 14–16]. However, in line with the World Health Organization (WHO) defining health as “a state of […] well-being and not merely the absence of disease” [17], salutogenic approaches for measuring mental health in patients with skin diseases are necessary in order to achieve a holistic understanding of the mental burden [12, 18, 19]. Accordingly, over the last years, studies exploring well-being in individuals with psoriasis have emerged [20–24]. In general, most studies comparing individuals with psoriasis to healthy controls found that well-being was impaired in this patient group, underlining the large mental burden of psoriasis [20, 21, 25]. However, there is increasing evidence pointing toward a more complex relationship between psoriasis and well-being, which seems to be mediated by the subjective perception of the symptoms rather than the objective course of the disease [21, 26, 27].

### Subjective well-being and depression in psoriasis

Recently, happiness has been discussed as a patient reported outcome with high subjective relevance for patients with chronic skin diseases [20]. Although different definitions exist [28], happiness is often conceptualized as subjective well-being [29]. According to Diener et al. [25], subjective well-being comprises an individual’s “cognitive and affective evaluation of life as a whole”. Following this approach, subjective well-being is high if one experiences frequent positive affect (PA), infrequent negative affect (NA) and a general satisfaction with life (SWL) [25, 30]. So far, only a few studies exploring subjective well-being in patients with psoriasis exist [23, 24, 31, 32], with only one study comprehensively assessing all three facets of subjective well-being in a comparably small group of 52 patients with psoriasis [20]. The results of the existing studies suggest that psoriasis patients are impaired in terms of PA but not SWL compared to healthy individuals [20, 23, 24]. For NA, results are inconsistent [20, 23, 31].

Depression, in contrast, is a well-researched and generally accepted comorbidity of psoriasis which has been studied in several systematic reviews and meta-analyses [33, 34]. As depression is characterized by the reduced capability to experience positive emotions [35], the finding of low PA in patients with psoriasis could be explained by the higher prevalence of depressive symptoms in this patient group, which raises the question whether exploring affective well-being independently of depression does in fact provide new insights on the mental burden of psoriasis. Studies exploring both subjective well-being and depression in individuals with psoriasis are needed in order to be able to distinguish these two constructs in this patient group.

### Aim of the study

The aim of this study was thus to assess both depression and subjective well-being (operationalized as PA, NA, and SWL) in a large and diverse sample of individuals affected by psoriasis, and to explore associations between depression, subjective well-being, and patient characteristics in this group.

## Materials and methods

### Study population and recruitment

For this cross-sectional study, an online survey among individuals affected by psoriasis in Germany was conducted from March to June 2019. The link to the online questionnaire was shared on Germany’s largest patients’ organization webpage for psoriasis (“Psoriasis Netz”, www.psoriasis-netz.de) and in the corresponding email newsletter and forum. In addition, the link was shared on other psoriasis-related Facebook pages (“Farbenhaut”, Technical University of Munich’s University Hospital and the de-stigmatization campaign “Bitte berühren” by the Professional Association of the German Dermatologists). Only individuals affected by psoriasis were eligible to participate, which was clearly stated on the first page of the online questionnaire and which had to be confirmed by the participants in the first question before they were able to continue with the rest of the questionnaire. Informed consent was obtained from all participants prior to inclusion. This study was conducted in accordance with the Declaration of Helsinki and was reviewed and approved by the local ethics committee of Technical University of Munich (reference 25/19 S).

### Study variables

The study questionnaire was developed by a multidisciplinary team including a dermatologist, a psychologist and two epidemiologists. It consisted of validated scales measuring subjective well-being and depression and additional questions on happiness in general and the evaluated impact of psoriasis. The survey was pre-tested by three psoriasis patients and, based on the resulting feedback, slightly modified. For the scales used in this study, internal consistency was considered “excellent” for Cronbach’s alpha values > 0.9 and “good” for Cronbach’s alpha values > 0.8 [36].

#### Subjective well-being

Following Diener et al. [25], subjective well-being was operationalized as *PA, NA* and *SWL.* PA and NA were measured using the validated German version of the Scale of Positive and Negative Experience (SPANE) [37, 38]. We chose the SPANE over the more frequently used Positive and Negative Affect Schedule (PANAS) [39], as it assesses a wider range of positive and negative emotions than the PANAS, which focusses on high arousal emotions, and as it is coherent with Diener and colleagues conceptualization of subjective well-being [37]. The SPANE consists of two subscales measuring PA and NA, respectively. Each subscale consists of six adjectives, e.g., “pleasant” and “positive” for PA and “unpleasant” and “negative” for NA). The respondents were asked to indicate how often they had felt the respective feelings over the past two weeks on a 5-point scale from 1 (“very rarely or never”) to 5 (“very often or always”). The two distinct subscales, showed excellent and good reliability in this study with Cronbach’s alphas of 0.93 and 0.86 for PA and NA, respectively. For each subscale, the items were averaged to form an index. *SWL* was measured using the German version of the Satisfaction With Life Scale (SWLS) [40, 41]. The scale consists of five items (e.g., “in most ways my life is close to my ideal”), each rated on a 7-point scale from 1 (“strongly disagree”) to 7 (“strongly agree”). The scale showed excellent reliability in this study with a Cronbach’s alpha of 0.91 and the items were averaged to form an index. As some researchers argue that happiness is not in fact a multidimensional but a unidimensional construct [29], we decided to also include a *heuristic measure of happiness*, meaning a single question asking participants for their overall happiness [20]. In order to achieve comparability with the general population, a single question from the European Social Survey was used: “Taking all things together, how happy would you say you are?” [42]. Respondents could give their answer on a 11-point scale from 0 (“extremely unhappy”) to 10 (“extremely happy”).

As an additional question on happiness in the context of psoriasis, the participants were asked for a *subjective evaluation of the impact of psoriasis on their own happiness* (“Do you think that your psoriasis has a negative impact on how happy you are?”—“No”, “Yes, a little”, “Yes, moderately”, “Yes, very much”).

#### Depression

The WHO-5 Well-Being Index [WHO-5, 43] is a validated screening questionnaire for *depression*. It consists of five statements (e.g., “My daily life has been filled with things that interest me”), which respondents rate on a 6-point scale from 0 (“at no time”) to 5 (“all of the time”). The items showed good reliability in this study with a Cronbach’s alpha of 0.87. Following the instructions, the values of the items were added, resulting in an overall score ranging from 0 to 25, with lower scores indicating a higher risk for depression. Using a cut-off of ≤ 7, the WHO-5 has shown good properties for the screening of major depression with a sensitivity of 94% and specificity of 78% [44]. Consequently, a score of ≤ 7 was considered a positive screening result for depression in this study.

#### Participants’ characteristics

As possible parameters associated with happiness, data on *age* (in years), *gender*, *years since first diagnosis*, and *current treatment status* (currently receiving treatment vs. not receiving treatment) were collected. Subjective *general disease severity* was measured as “mild”, “moderate”, “severe”. In addition, data on current disease severity were collected using the same given options (“mild”, “moderate”, “severe”). Based on general and current disease severity, new dichotomous variables indicating current phases of *relative improvement* or *relative deterioration* compared to general disease severity were derived.

### Analysis

Prior to the analysis, data were checked for completeness (at least 80% of questions on happiness answered) and plausibility (e.g., data were considered implausible if the participants indicated a higher number of years since first diagnosis than age). As only very few participants did not meet our criteria for completeness and plausibility (*n* = 8), these cases were excluded from all further analysis rather than using imputation in order to keep the analysis as simple as possible. The remaining data were analyzed descriptively. Pearson’s correlations were calculated in order to explore associations between the examined variables. Correlations were considered moderate for *r* > 0.5 and strong for *r* > 0.7 [45]. To further differentiate the four constructs PA, NA, SWL, and depression, exploratory factor analysis using Promin Rotation, an oblique rotation method appropriate for correlated variables, was conducted after checking the respective requirements were fulfilled. As all variables were measured on at least 5-step scales (with equal distances between the answer options and numbers suggesting equal steps between the categories), variables were treated as quasi-metric and Pearson’s correlations were used for factor analysis. Following Guadagnoli and Velicer [46], factor loadings of 0.4 and higher were considered stable, which is why all smaller coefficients were suppressed. Factor retention was determined using parallel analysis [47]. However, as the retention of three factors, which was the number of factors suggested by parallel analysis, did not result in stable factor loadings for all examined items, we additionally conducted the analysis retaining one more factor, which was in line with the theoretical assumption of four differing constructs of PA, NA, SWL, and depression.

Means of PA, NA, heuristic happiness, SWL and WHO-5 were compared to norm data [41, 48, 49] or, in case of PA and NA, to data of a validation study [38] as norm data were not available. For both SWL and WHO-5, norm data had been collected in representative samples of the German general population (with the assistance of a demographic consulting company (SWL: mean age 48.9 ± 18.3 years, 52.2% women; WHO-5: mean age 48.3 years, SD not indicated for the overall sample, 52.7% women) [41, 48]. For heuristic happiness, norm data for the German general population were collected within the European Social Survey (mean age 48.2 ± 18.1 years, 49.9% women) [49]. For PA and NA, the data of the German validation study among a total of 498 participants recruited in university lectures (*n* = 264) and via mailing lists/social media (*n *= 234) were used as reference data (mean age not indicated, 18.5% < 20 years, 47.8% 20–29 years, 16.3% 30–39 years, 7.7% 40–49 years, 5.3% 50–59 years, 4.5% > 59 years, 75.1% women) [38]. As for PA, NA, and heuristic happiness the datasets of the reference samples were accessible, the group comparisons for these variables were conducted using ANCOVA and planned contrasts while controlling for sex and age. Adjusted means (m^a^) are reported. For SWL and WHO-5, the datasets of the reference populations could not be retrieved, which is why analyses were conducted stratified for age and sex, using Student’s *t*-tests. The age groups for these analyses were chosen to match the age groups reported in the respective reference samples. As Student’s *t*-tests and ANCOVA have been shown to provide robust results even when normality and equal variance assumptions are violated [50–52], the analyses were conducted without prior verification of these assumptions.

Parameters associated with subjective well-being and a positive screening for depression were identified using multiple linear regression models and binary logistic regression, respectively. In all regression models, age, gender, years since first diagnosis, general subjective disease severity, improvement or deterioration of skin condition and current treatment status were entered as independent variables. As a result, adjusted raw (B) and standardized regression coefficients (*β*) and Odds Ratios (OR) as well as corresponding 95%-confidence intervals (CIs) and the percentage of variance explained by each model (adjusted R^2^) are reported. As depression is characterized by the inability to feel happy [35], we conducted additional sub-group analyses in participants who did not receive a positive screening result for depression in order to explore differential findings for affective well-being which are not explained by the presence of depression.

The level of significance was set at *α* = 0.05 for all analyses. All statistical analyses except exploratory factor analysis were conducted using IBM SPSS Statistics Version 24 (IBM Corporation, Armonk, NY, USA). Exploratory factor analysis was conducted using FACTOR software [53].

## Results

In total, 730 participants completed the survey, with most users having participated via “Psoriasis Netz” (95%). Of those, eight were excluded prior to analysis due to insufficient (*n* = 3) or implausible data (e.g., higher number of years since first diagnosis than age; *n* = 5). Consequently, the data of 722 participants were analyzed. The mean age was 45.8 years with a standard deviation (SD) of 13.4 years and a range of 12–85 years (Table 1). 62.7% of participants were women. Almost all participants (98.6%) reported having been diagnosed with psoriasis by a medical doctor, and only ten participants (1.4%) had been given the diagnosis by an alternative practitioner or had self-diagnosed psoriasis. On average, the participants had been living with psoriasis for 20.6 years (SD = 14.5 years, range < 1–68). The majority of participants (55.7%) judged their psoriasis to be generally moderate, 34.3% severe, and only 10% mild. When asking for current disease severity, 24% of participants indicated mild, 52.8% moderate, and 23.3% severe. Comparing self-reported general and current disease severity, 31.7% of participants were in a phase of relative improvement, and 14.4% of relative deterioration. One out of four participants (24.8%) was not currently receiving medical treatment for psoriasis. Of those who were receiving treatment, the majority (82.1%) were treated by a dermatologist, 23.3% by a rheumatologist, 21.5% by a general physician, and 3.2% received alternative care (e.g., by an alternative practitioner).

**Table 1:** Baseline patients’ characteristics

Patients’ characteristics	*N* = 722
Age in years (mean ± SD, range)	48.8 ± 13.4, 12–85
Gender	
- Male	269 (37.3%)
- Female	453 (62.7%)
Diagnosis	
- By medical doctor	712 (98.6%)
- By alternative practitioner or self-diagnosis	10 (1.4%)
Years since first diagnosis (mean ± SD, range)	20.6 ± 14.5, 0–68
General disease severity	
- Mild	72 (10%)
- Moderate	402 (55.7%)
- Severe	248 (23.3%)
Current disease severity	
- Mild	173 (24%)
- Moderate	381 (52.8%)
- Severe	168 (23.3%)
Phase of relative improvement of skin condition	229 (31.7%)
Phase of relative deterioration of skin condition	104 (14.4%)
Current treatment^a^	
- Dermatologist	446 (61.8%)
- Rheumatologist	127 (17.6%)
- General practitioner	117 (16.2%)
- Alternative care	23 (3.2%)
- No current treatment	179 (24.8%)

^a^Multiple answers were possible

### Subjective well-being and depression

**Fig. 1:**
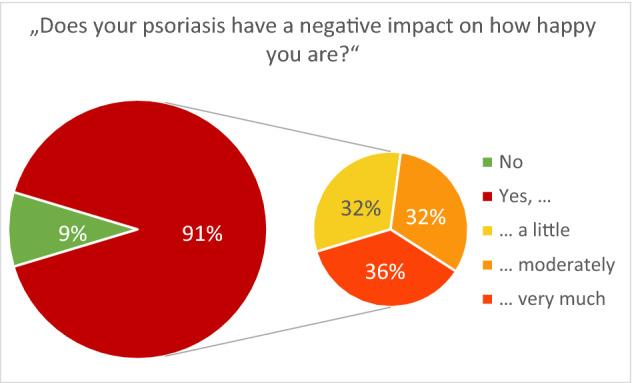
Subjective evaluation of the impact of psoriasis on patients’ happiness. *N* = 721, one missing case

The variables intercorrelated moderately to strongly, with the strongest correlation observed for PA and heuristic happiness (*r* = 0.73, *p* < 0.001; Table 2) and the weakest correlation observed for SWL and NA (*r* = − 0.56, *p* < 0.001). 40.3% of participants had a positive screening result for depression (41.9% of women and 37.5% of men). Nine out of ten participants (90.3%) stated that psoriasis had a negative impact on how happy they were (28.8% a little, 29% moderately, and 32.9% very much), and only 9.3% of participants stated that their psoriasis did not affect their happiness at all (Fig. 1).

**Table 2:** Correlation matrix

	PA	NA	SWL	Heuristic happiness	WHO-5 well-being index
PA	1				
NA	− 0.669***	1			
SWL	0.652***	− 0.564***	1		
Heuristic happiness	0.728***	− 0.588***	0.629***	1	
WHO-5 well-being index	0.687***	− 0.651***	0.630***	0.591***	1

Pearson’s correlation coefficients are displayed

Asterisks indicate significant correlations; *p < 0.05, **p < 0.01, ***p < 0.001

**Fig. 2:**
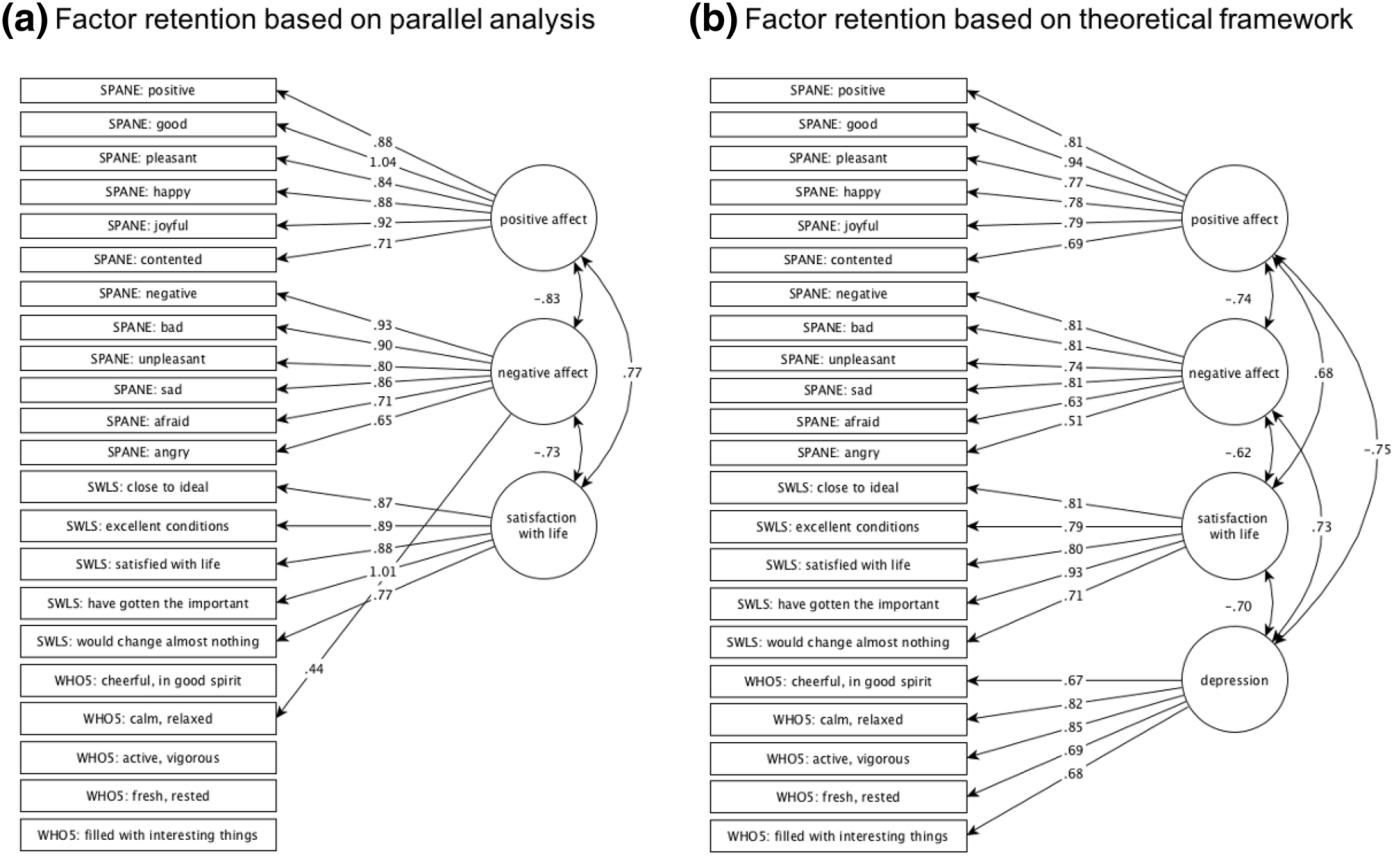
Factor loadings of the examined variables of the SPANE, the SWLS, and the WHO-5 **a** on the three factors retained based on parallel analysis **b** on four factors corresponding to the four constructs PA, NA, SWL, and depression. Arrows between factors show inter-factor correlations

Using exploratory factor analysis and parallel analysis for factor retention, three factors were identified (Fig. 2a). The first factor corresponded to the PA items of the SPANE, the second corresponded to the items of the SWLS, and the third factor corresponded to the NA items of the SPANE, with one item of the WHO-5 loading on the same factor. The remaining items of the WHO-5 did not show stable factor loadings on any of the three factors. When adding one more factor to be extracted, all items of SPANE, SWLS, and WHO-5 loaded on four separate factors corresponding to the four constructs PA, NA, SWL and depression (Fig. 2b).

Descriptive statistics of PA, NA, SWL, heuristic happiness, and WHO-5 are displayed in Table 3.

**Table 3:** Descriptive statistics of the examined variables in the study sample and the respective reference samples

	Mean ± SD	Adjusted mean/means per group ± SD	Adjusted mean/means per group ± SD (reference)	Significance^a^
PA	2.96 ± 0.89	2.98	3.10	0.003
NA	2.83 ± 0.89	2.87	2.88	0.767
Heuristic happiness	5.32 ± 2.37	6.34	7.26	<0 .001
SWL	4.27 ± 1.43			
- 14–24 years		19.63^b^ ± 6.24	24.77^c^ ± 6.11	< 0.001
- 15–34 years		21.87^b^ ± 7.47	25.89^c^ ± 6.34	< 0.001
- 35–44 years		20.77^b^ ± 7.66	25.01^c^ ± 6.37	< 0.001
- 45–54 years		20.80^b^ ± 6.79	24.43^c^ ± 6.81	< 0.001
- 55–64 years		21.28^b^ ± 7.28	24.15^c^ ± 6.32	< 0.001
- 65–74 years		23.73^b^ ± 6.13	25.30^c^ ± 5.52	0.059
- > 74 years		24.56^b^ ± 3.78	25.06^c^ ± 5.93	0.802
- Men		20.86^b^ ± 7.40	25.12 ± 6.32	<0 .001
- Women		21.62^b^ ± 7.05	24.67 ± 6.20	< 0.001
WHO-5 well-being index	9.86 ± 5.27			
- < 41 years		9.73 ± 4.96	18.36 ± 4.80	< 0.001
- 41–60 years		9.41 ± 5.30	17.49 ± 4.88	< 0.001
- > 60 years		11.9 ± 5.65	16.70 ± 5.13	< 0.001
- Men		10.34 ± 5.61	18.15 ± 4.90	< 0.001
- Women		9.58 ± 5.05	17.07 ± 4.98	< 0.001

^a^ANCOVA and Student’s *t*-tests, respectively

^b^For stratified analysis items were summed up (instead of averaged) in order to allow for comparison with reference data

^c^Calculated based on weighted means for men and women

### Comparison with reference populations

Controlling for age and sex, participants in this study reported significantly less PA than the German validation sample (*m*^a^ = 2.98 vs. 3.10, *p* = 0.003) [38], but did not differ from them in terms of NA (*m*^a^ = 2.87 vs. 2.88, *p* = 0.767; Table 3). Furthermore, controlling for age and sex, participants in this study judged themselves less happy than the general population (*m*^a^ = 6.34 vs. 7.26, *p* < 0.0001). Stratifying for age and sex, participants of this study over all age groups and both men and women scored significantly lower on the WHO-5 than the general population (all *p* < 0.001). Furthermore, stratified analyses showed that participants under 65 years in this sample were less satisfied with their lives than the general population under 65 years (all *p* < 0.001), while participants of 65 years and older did not differ from their peers in terms of SWL (*p* > 0.05). Taking all age groups together, both men and women in this study reported lower SWL than men and women of the general population (both *p* < 0.0001).

### Factors associated with subjective well-being and depression

#### PA

**Fig. 3:**
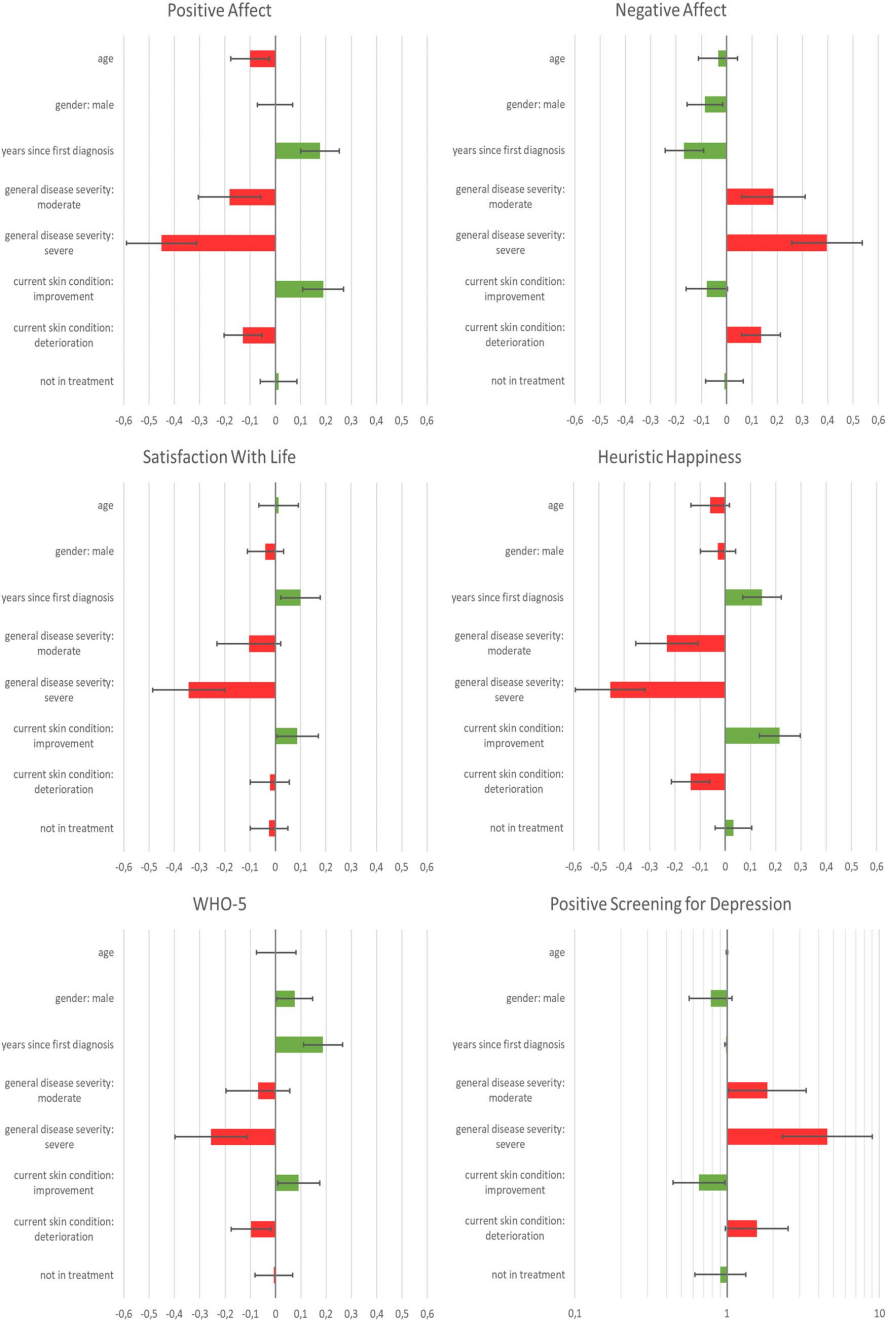
Determinants of happiness and depression in 722 individuals with psoriasis. Adjusted standardized regression coefficients (for positive screening of depression: OR on log scale) with corresponding confidence intervals are given. Factors associated with higher well-being are displayed in green and those associated with lower well-being are displayed in red. Happiness was operationalized as subjective well-being, consisting of PA, NA, and SWL, and as a heuristic evaluation of happiness. A screening result for depression was considered positive if a participant scored ≤ 7 in the WHO-5. Asterisks indicate significant correlations. *p < 0.05, **p < 0.01, ***p < 0.001. (Color figure online)

Higher age [B = − 0.01, CI (− 0.12; − 0.002); *β* = − 0.10, CI (− 0.18; − 0.03)], moderate [B = − 0.33, CI (− 0.55; − 0.11); *β* = − 0.18, CI (− 0.31; − 0.06)] and severe [B = − 0.85, CI (− 1.11; − 0.59); *β* = − 0.45, CI (− 0.59; − 0.31)] general disease severity as compared to mild disease severity and deterioration of skin condition [B = − 0.32, CI (− 0.53; − 0.13); *β* = − 0.13, CI (− 0.2; − 0.05)] were associated with lower levels of PA (Fig. 3). In contrast, a higher number of years since the first diagnosis [B = 0.11, CI (0.01; 0.02); *β* = 0.18, CI (0.10; 0.25)] and improvement of skin condition [B = 0.36, CI (0.21; 0.52); *β* = 0.06, CI (0.11; 0.27)] were associated with higher PA. The full model explained 10.7% of variance in PA (*p* < 0.001). When including only patients without depression, the same parameters were associated with PA, but the model explained only 7.5% of variance (*p* < 0.001).

#### NA

NA was higher in participants with moderate [B = 0.33, CI (0.11; 0.55); *β* = 0.19, CI (0.06; 0.31)] and severe [B = 0.74, CI (0.48; 1); *β* = 0.40, CI (0.26; 0.54)] general disease severity and those who were currently in a phase of deterioration of the skin condition [B = 0.34, CI (0.15; 0.54); *β* = 0.14, CI (0.06; 0.21); Fig. 3]. In contrast, being male [B = − 0.16, CI (− 0.29; − 0.03); *β* = − 0.09, CI (− 0.16; − 0.02)] and living with psoriasis for a longer time [B = − 0.01, CI (− 0.02; − 0.006); *β* = − 0.17, CI (− 0.24; − 0.09)] were associated with less NA. The full model explained 9.1% of variance in NA (*p* < 0.001). When including only patients without depression, the association remained significant for moderate and severe general disease severity and years since the diagnosis of psoriasis (*p* = 0.008; *p* = 0.001; *p* < 0.001, respectively), but the associations with gender and deterioration of the skin did not reach significance anymore (*p* = 0.071 and *p* = 0.085, respectively). Similar to PA, the model explained slightly less variance (7.6%, *p* < 0.001) in participants without depression compared to the whole sample.

#### SWL

Severe general disease severity was associated with lower levels of SWL [B = − 1.04, CI (− 1.47; − 0.61); *β* = − 0.34, CI (− 0.49; − 0.20); Fig. 3]. More years passed since the first diagnosis [B = 0.01, CI 0.002; 0.02); *β* = 0.10, CI (0.02; 0.18)] and current improvement of skin condition [B = 0.27, CI (0.01; 0.52); *β* = 0.09, CI (0.00; 0.17)] were associated with higher SWL. The full model explained 5.5% of variance (*p* < 0.001) in SWL. When excluding participants with depression, only female gender was significantly associated with SWL (*p* = 0.049). Overall, the model did not show a good fit for this sub-sample (adjusted R^2^ = 0.013, *p* = 0.099).

#### Heuristic happiness

Patients who were in general moderately [B = − 1.11, CI (− 0.1.7; − 0.51); *β* = − 0.24, CI (− 0.36; − 0.11)] or severely [B = − 2.28; CI (− 2.97; − 1.59); *β* = − 0.46, CI (− 0.60; − 0.32)] affected or who were currently in a phase of deterioration [B = 1.1, CI (0.69; 1.51); *β* = − 0.14, CI (− 0.22; − 0.07)] judged themselves to be less happy (Fig. 3). In contrast, those who had been living with psoriasis for more years [B = 0.02, CI (0.01; 0.04]; *β* = 0.14, CI (0.07; 0.22)] or who were in a current phase of improvement of skin condition [B = 1.1, CI (0.69; 1.51); *β* = 0.22, CI (0.14; 0.30)] judged themselves to be happier. The full model explained 10.2% of variance in heuristic happiness. All parameters associated with heuristic happiness remained significant when excluding patients with depression, and the model explained 6.9% of variance in heuristic happiness (*p* < 0.001).

#### Depression

Participants with severe psoriasis [B = − 2.84, CI (− 4.41; − 1.27); *β* = − 0.26, CI (− 0.40; − 0.11)] and currently going through a phase of deterioration [B = − 1.46, CI (− 2.62; − 0.3); *β* = − 0.10, CI (− 0.18; − 0.02)] scored lower in the WHO-5 questionnaire, which indicates higher levels of depressive tendencies (Fig. 3). In contrast, men [B = 0.82, CI (0.04; 1.6); *β* = 0.08, CI (0.01; 0.15)], participants living with psoriasis for a longer time [B = 0.07, CI (0.04; 0.1); *β* = 0.19, CI (0.11; 0.27)] and those currently experiencing improvement of skin condition [B = 1.04, CI (0.11; 1.98); *β* = 0.09, CI (0.01; 0.18)] scored higher in the WHO-5. The full model explained 6.6% of variance in the WHO-5 score (*p* < 0.001). Moderately affected participants had a 1.8-fold [CI (1.02; 3.32)] and severely affected participants even had a 4.6-fold [CI (2.31; 9.03)] chance of being screened positive for depression compared to mildly affected participants (Fig. 3). Living with psoriasis for a year longer [OR 0.98, CI (0.97; 0.99)] and current improvement of skin condition [OR 0.65, CI (0.44; 0.97)] were both associated with a decreased chance of a positive screening for depression. The full model explained 8.6% of variance in screening results (*p* < 0.001).

## Discussion

This study examined both subjective well-being and depression in patients with psoriasis, following the WHO’s demand for a holistic approach of measuring mental health in this patient group [12]. In a nationwide sample of 722 individuals affected by psoriasis in Germany, subjective well-being and more precisely PA and SWL were low compared to the general population or healthy reference populations, and 40% of participants were screened positive for depression. The main risk factor for low subjective well-being and depression was general disease severity, but also deterioration of the skin was negatively associated with the affective component of subjective well-being, while phases of improvement of skin condition were associated with more PA and higher SWL. Additionally, the longer participants had been living with psoriasis, the higher was their subjective well-being and the less depressed they were.

### Differentiating depression and well-being

Due to the simultaneous assessment of depression and subjective well-being, this study allowed to examine the association between these constructs in individuals affected with psoriasis. As depression, among other symptoms, is characterized by the absence of PA [35], the constructs of depression and subjective well-being/positive affect are bound to overlap per definition, which is reflected by overlapping items of the respective measurement tools (especially question 1 of the WHO-5 and the PA items of the SPANE) [54]. However, depression is also characterized by other symptoms such as lack of energy and decreased activity which are not covered by PA/subjective well-being. Accordingly, exploratory factor analysis revealed that all items of SPANE, SWLS and WHO-5 loaded on four different factors corresponding to PA, NA, SWL and depression, supporting the methodological approach of differentiating depression and subjective well-being. This is especially interesting as the empirical differentiation of subjective well-being and depression, which has been successfully shown in some studies [55], has proven difficult in others [56]. The results of this study are in line with the postulate of the WHO that well-being is more than the absence of illbeing (see also Lukat et al. [57]). Thereby, they underline the WHO’s demand to including measures of well-being in clinical research and practice in individuals with psoriasis in order to achieve a more comprehensive understanding of the mental burden of this disease [12].

### Subjective and objective disease severity

Subjective general disease severity was associated with all facets of subjective well-being in this study. In contrast, two previous studies have only found associations between disease severity and single facets of subjective well-being (NA: Martin-Brufau et al. [23]; PA: Schuster et al. [20], respectively). These inconsistencies in the findings might be owed to the different modalities of assessing disease severity employed in the different studies: Previous studies have shown that the subjective perception of the symptoms might be more important for individual well-being than the objective course of the disease [21, 26, 27]. While the two mentioned studies used either physician assessed disease severity [20] or a validated self-assessment tool for disease severity [23], a very subjective approach was used in the present study, with participants rating their disease severity as “mild”, “moderate” or “severe”, This approach might explain why the associations between well-being and disease severity were especially pronounced in this study. More research is needed in order to shed light on the (different?) roles of subjective and objective disease severity for well-being and mental health. An alternative explanation could be that the small sample size of 52 patients in the study of Schuster et al. [20] prevented the detection of associations between disease severity and NA and SWL observed in this study.

### General and current disease severity

Interestingly, high general disease severity remained strongly associated with low subjective well-being and depression even when current skin condition was taken into account. While improvement of the skin was associated with higher well-being, the negative association between general severe disease severity and well-being was more pronounced. These findings implicate that by relieving skin symptoms, physicians might be able to help patients feel happier and, as reported before [15, 16], reduce the risk for depression. However, especially in more severe forms of psoriasis, well-being might remain low even if the symptoms improve. This supports the idea that documenting the PeakPASI, meaning the highest PASI ever recorded per patient, might provide additional information about the mental burden of psoriasis compared to just referring to the “snapshot” PASI at a single visit [58].

### The role of online recruitment

The participants in this sample scored lower than the German norm in SWL and reported less PA than a healthy reference sample. While the findings of impaired PA, but not increased NA, is in line with previous findings [20], the reported values for PA, NA and SWL were even more unfavorable than in a previous study among patients with psoriasis [20]. Also, we found a high rate of positive screening for depression of 40%. An explanation for these findings could be that the recruitment strategy might have led to a sample which was especially impaired in terms of well-being and depression. This would suggest that especially unhappy individuals turn to (online offers of) self-help organizations like the one used for recruitment in this study, which would underline the great importance these entities have for the comprehensive treatment of individuals with psoriasis. In contrast, however, it is also possible that, due to the anonymity, the participants in this online survey were more honest and open about their well-being than in previous studies, which have mostly taken place in medical settings [20, 24, 32]. Thus, social desirability might have led to an underestimation of happiness and well-being in previous studies.

### Limitations

The results of this study are subject to several limitations. First, as the survey was conducted online, selection bias must be considered, which was already discussed in the above paragraph. Second, while all participants had to actively indicate that they were indeed affected by psoriasis prior to taking part in the survey, the online setting did not allow to confirm the diagnosis. Third, due to time limitations in the survey, which was designed to take only a few minutes to complete in order to achieve a high number of participants, disease severity was not assessed using validated scales or clinical scores but as “mild”, “moderate” and “severe”. Future studies should explore whether the findings of this study can be replicated when using validated scores for the assessment of subjective or even objective disease severity like PASI. Also, as this is a cross-sectional study, we could not determine causality but only association. Finally, future studies should explore the role of personality traits and other possible moderator variables such as marital status for the examined associations, as they have not been considered in this study.

### Strengths

This study followed a holistic approach of assessing mental health in individuals with psoriasis by measuring not only depression, but also subjective well-being in a large sample of affected individuals. Thus, the study does not only contribute to the growing body of research exploring well-being in psoriasis healthcare, it furthermore adds empirical evidence supporting the differentiation of depression and well-being as two related constructs. Furthermore, the study was designed in a way to not only include patients in the medical setting, but also affected individuals who were not currently part of the health care system, which is in line with the WHO’s demand for “people centered care” instead of “patient centered care” [59]. Thus, by looking at people with psoriasis in general instead of focusing on patients only (= people who are currently receiving medical care), the study provides a more comprehensive picture of mental health in individuals with psoriasis regardless of treatment status than prior studies which were mostly conducted in medical settings. Finally, the online setting of this study allowed the participants to fill in the questionnaire in an anonymous setting, which might help to reduce social desirability bias as compared to paper-based questionnaires at doctors’ offices or clinics which are frequently employed in epidemiological research.

### Future research

Interventions targeting NA (especially stress) have been successfully conducted in patients with psoriasis [60, 61]. As this and previous studies have shown that PA seems to be especially impaired in patients with psoriasis [20], and as PA has been linked to several desirable health outcomes like better immune reactions, faster skin barrier recovery, and better cardiovascular health [62, 63], future research should evaluate whether interventions targeting PA could be also or maybe even more beneficial for improving the patients’ well-being beyond the treatment of skin symptoms. Furthermore, future research should explore the mechanisms behind the presumably beneficial effect of skin treatment on well-being, as it could be moderated and potentially improved by additional factors such as time to treatment response [26].

### Conclusion

In this large sample of 722 patients with psoriasis, participants reported lower subjective well-being than the German general population or healthy reference populations. General disease severity was associated with both low subjective well-being and depression, even in phases of improvement of the skin condition. While improvement of skin condition, which can be achieved by an effective treatment of skin symptoms, was associated with higher well-being and less depression, the negative associations between severe general disease severity and well-being and depression, respectively, were even more pronounced. As subjective well-being and depression have been identified as two differential constructs in this study, measures of well-being should be increasingly incorporated in psoriasis health care.

## Data Availability

Data are available at http://dx.doi.org/10.23668/psycharchives.4451.

## Code availability

Code is available upon request.
